# Human MSC-Derived Exosomes Reduce Cellular Senescence in Renal Epithelial Cells

**DOI:** 10.3390/ijms222413562

**Published:** 2021-12-17

**Authors:** Chieh Ming Liao, Tianjiao Luo, Juliane von der Ohe, Blanca de Juan Mora, Roland Schmitt, Ralf Hass

**Affiliations:** 1 Department of Nephrology and Hypertension, Hannover Medical School, 30625 Hannover, Germany; Liao.Chieh@mh-hannover.de (C.M.L.); deJuanMora.Blanca@mh-hannover.de (B.d.J.M.); Schmitt.Roland@mh-hannover.de (R.S.); 2 Biochemistry and Tumor Biology Lab, Department of Gynecology and Obstetrics, Hannover Medical School, 30625 Hannover, Germany; luo_tj@yeah.net (T.L.); Ohe.Juliane.von.der@mh-hannover.de (J.v.d.O.)

**Keywords:** senescence, exosome, MSC, kidney, PTEC, aging, SASP

## Abstract

Cellular senescence of renal tubular cells is associated with chronic diseases and age-related kidney disorders. Therapies to antagonize senescence are, therefore, explored as novel approaches in nephropathy. Exosomes derived from human mesenchymal stroma-/stem-like cells (MSC) entail the transfer of multiple bioactive molecules, exhibiting profound regenerative potential in various tissues, including therapeutic effects in kidney diseases. Here, we first demonstrate that exosomes promote proliferation and reduce senescence in aged MSC cultures. For potential therapeutic perspectives in organ rejuvenation, we used MSC-derived exosomes to antagonize senescence in murine kidney primary tubular epithelial cells (PTEC). Exosome treatment efficiently reduced senescence while diminishing the transcription of senescence markers and senescence-associated secretory phenotype (SASP) factors. Concomitantly, we observed less DNA damage foci and more proliferating cells. These data provide new information regarding the therapeutic property of MSC exosomes in the development of renal senescence, suggesting a contribution to a new chapter of regenerative vehicles in senotherapy.

## 1. Introduction

As the population ages, challenges of age-associated diseases are steadily growing and pose an enormous burden to public health. One of the organs most affected by age-associated changes is the kidney. Aging of the kidney is characterized by a gradual loss of resistance to stress and a decreased capacity to repair, leading to a dramatically increased prevalence of chronic kidney disease (CKD) among the elderly [1]. Patients with CKD are susceptible to accelerated aging, which can be associated with the appearance of further pathologies, including cardiovascular diseases with systemic inflammatory atherosclerosis [2]. Recent evidence indicates that antagonizing the phenomenon of cellular senescence is a novel therapeutic option to improve healthy kidney aging and to foster intrinsic repair mechanisms [3,4,5,6].

Cellular senescence is a specialized cell state of permanent cell cycle arrest caused by the accumulation of cellular damage due to a variety of stressors, such as telomere shortening, DNA damage, oxidative stress, and activation of oncoproteins [7,8,9]. Senescent cells interfere with the functionality of tissues and organs due to their inability to divide, an accompanying loss of specialized functions and the secretion of active biomolecules, which trigger inflammation and cell dysfunction, summarized as the senescence-associated secretory phenotype (SASP) [10].

Senescent cells and their damage-associated products accumulate in multiple tissues with progressive age. Accordingly, correlations between cellular senescence and chronic diseases have been reported in multiple organs [8,11,12,13].

A variety of different mechanisms can promote senescence. These include the generation of reactive oxygen species (ROS) during premature senescence. Besides the induction of ROS by radiation exposure or pharmacologic agents (e.g., anthracyclins during chemotherapy), many ROS-mediated pathologies are known to promote ‘stress or aberrant signaling-inducing senescence’ (STASIS) [14]. Another mechanism, also termed replicative senescence, is triggered by the progressive erosion and subsequent dysfunction of telomeres during the proliferative cell cycle [15]. Shortened telomere signaling in replicative senescence may be defined by the Hayflick limit or mortality stage-1 [16,17]. Therefore, the cells exhibit characteristics of a senescent phenotype, including small telomere restriction fragment length, a viable G_0′_-like cell cycle arrest, and the expression of senescence-associated beta-galactosidase (SA-β-gal). However, previous studies in human mammary epithelial cells have demonstrated potential escape mechanisms from a senescent phenotype [18,19].

In the kidney, the accumulation of senescent cells is thought to contribute to renal fibrosis, diabetic nephropathy, severe acute kidney injury and decay in renal function [20]. As the major cell type in the kidney, tubular epithelial cells play a key role in maintaining electrolyte balance and fluid homeostasis, whereas tubular senescence has been linked to aging and nephropathy [4,11]. Several recent studies have indicated that the removal of senescent tubular cells in the kidney by transgenic or pharmaceutical approaches reduced features of tissue aging and efficiently ameliorated glomerulosclerosis, inflammation and renal function [3,4,5,6,21]. Therefore, the development of senotherapeutics represents a rapidly expanding field. Senotherapeutics can be classified into senolytics and senomorphics [22]. Senolytics represent pharmaceutical approaches to specifically target senescent cells by selectively eliminating senescent cells with minimal harm to non-senescenct cells [23]. Since 2015, several senolytics have been discovered, and some of these compounds have already progressed into clinical phase I and II trials [24]. Alternatively, senomorphics can modulate the morphology and certain functions of senescent cells while leaving them alive [22].

Cell-free natural products, such as extracellular vehicles (EVs) including exosomes from human mesenchymal stroma-/stem-like cells (MSC), are able to improve regenerative mechanisms and putatively function as senotherapeutic agents [25]. Their properties may include the promotion of reversible senescence as part of a retrodifferentiation program and rejuvenation [26,27]. Among others, MSC can promote repair activities by driving immune modulation, and neovascularization upon recruitment to damaged sites, where they release trophic factors and exosomes with curative cargo [28,29]. Thus, MSC-derived exosomes represent cell-free vehicles that entail significant therapeutic potential. These nanoparticles bridge the communication between cells by delivering their content of bioactive molecules, including lipids, proteins, mRNAs, transfer RNA, long noncoding RNAs, microRNAs and mitochondrial DNA [30]. The reported beneficial and therapeutic effects of MSC-derived exosomes include damage protection, the reduction in cancer progression, and immune modulation [31,32,33]. In the present study, we explored the therapeutic potential of human MSC-derived exosomes to reduce progressive growth-arrest and senescence in high passages of MSC primary cultures and, similarly, to combat cellular senescence in kidney tubular cells.

## 2. Results

Exosomes represent small particles released from various cell types that shuttle a plethora of different proteins, metabolites, DNA fragments, and various RNAs (e.g., mRNAs, circRNAs, tRNAs, long non-coding RNAs, and regulatory microRNAs) [34,35,36,37,38] to target and alter the functionality of recipient cells [39,40,41]. According to an optimized yield of MSC-derived exosome production in [42], the collection of exosomes was systematically performed from serum-free stem cell cultures after 24 h. The different exosome preparations in relation to the appropriate cell numbers in the cultures were examined by nanotracking analysis (NTA) for the average sizes and amount of exosomes (Table 1). Based on the results of the BCA protein assay, the protein amount per exosome was calculated (Table 1).

**Figure 1:**
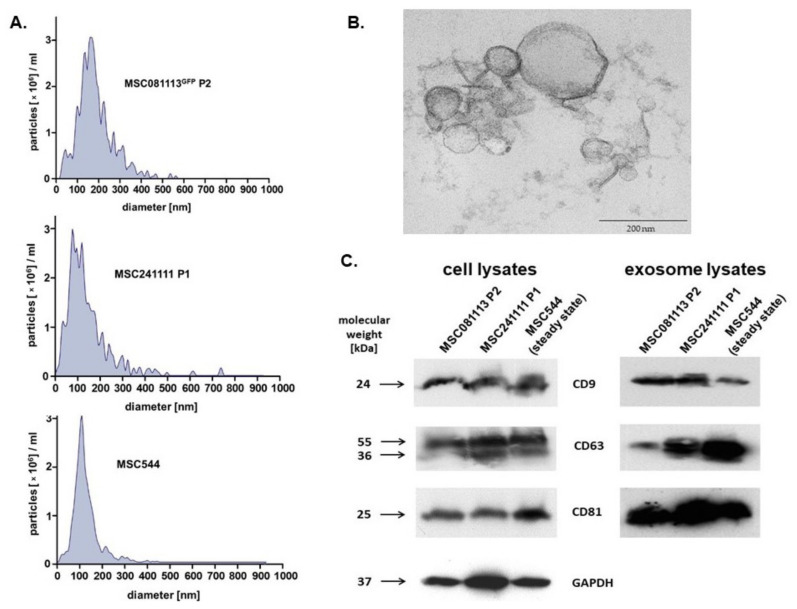
(**A**) Histograms of isolated vesicles from 24 h serum-free conditioned media of three different MSC populations were obtained after analysis in a ZetaView PMX120 NTA in scatter mode. (**B**) The vesicles were fixed in glutaraldehyde, embedded in Araldit CY212 (PLANO, Wetzlar, Germany), and analyzed with a TEM Morgagni 268 (FEI Company, Eindhoven, The Netherlands), demonstrating MSC544-derived exosomes as a representative image. The bar represents 200 nm. (**C**) The three different exosome-producing MSC populations (**left** panel) and corresponding MSC-derived vesicles (**right** panel) were homogenized, and protein was quantified by the BCA method. Aliquots of 40 µg protein from cell homogenates and 20 µg protein from the exosome preparations, respectively, were separated by SDS polyacrylamide gel electrophoresis and analyzed for the presence of exosome-specific tetraspanin molecules. Expression of GAPDH in the cell homogenate immunoblot (**left** panel) served as a loading control.

The data in Table 1 demonstrated certain variability in exosome yield among the different individual MSC donors, which is consistent with previous studies [42]. Further characterization of the different exosome preparations was performed by exosome size distribution, transmission electron microscopy (TEM), and immunoblots (Figure 1).

Size distribution histograms of isolated vesicles from 24 h serum-free conditioned media were obtained from young human MSC081113^GFP^ P2 (Figure 1A, upper panel), from young human MSC241111 P1 (Figure 1A, middle panel), and from permanently growing human MSC544 in steady state (Figure 1A, lower panel).

The vesicles from all three different MSC populations demonstrated typical size distributions of exosomes, respectively. The TEM analysis of these membranous vesicles further substantiated the size and morphology of previous studies [42,43] with a representative image of MSC544-derived exosomes (Figure 1B).

The presence of the tetraspanins CD9, CD63, and CD81 represents core markers for the identification of exosomes. Thus, immunoblot analyses of cellular protein homogenates from the producing MSC populations (Figure 1C, left panel) and from the corresponding MSC-derived exosome homogenates (Figure 1C, right panel) revealed altered expression levels of the 24 kDa CD9 protein, the 30 to 60 kDa glycosylated form of CD63, and the 25 kDa CD81 in all of the investigated samples (Figure 1).

**Figure 2:**
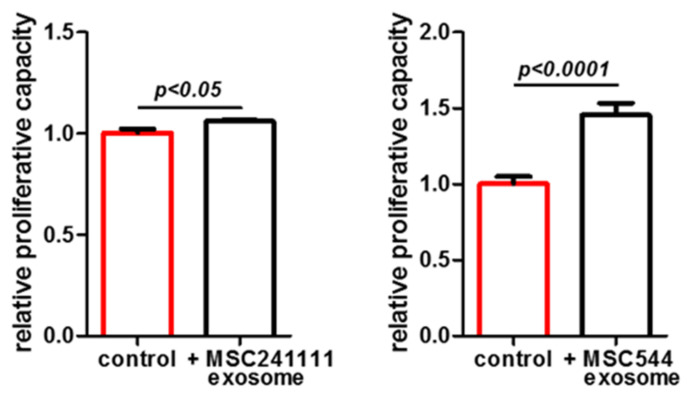
Proliferative effects of exosomes isolated from two different MSC donors were tested in a growth-arrested and senescent MSC population. About 5000 cells/well of the senescent UC-MSC150819 in passage 12 (P12) were plated in a 24-well plate with 1 mL of MSC growth medium, respectively. The cells in each well were stimulated either with 50 µL PBS (control) or with 50 µL of PBS-resuspended exosomes (from MSC241111 P1 or from MSC544) containing 1.3 × 10^9^ exosomes (=ratio of 2.6 × 10^5^ exosomes/cell). The population bars expressing higher senescence are marked in red. The cell numbers in the wells were counted after 72 h. Data represent the mean ± SEM of 3 replicates (*n* = 3), and significance was calculated by unpaired *t*-test.

These findings demonstrated that all three MSC populations produce and release EVs that display the size and morphology and simultaneously carry the tetraspanin molecules typical for exosomes. While previous work has suggested the development of a growth-arrested and senescent phenotype in primary MSC cultures beyond P10 [44], further studies were performed to test the potential effects of different exosome preparations on senescent MSC cultures (Figure 2).

**Figure 3:**
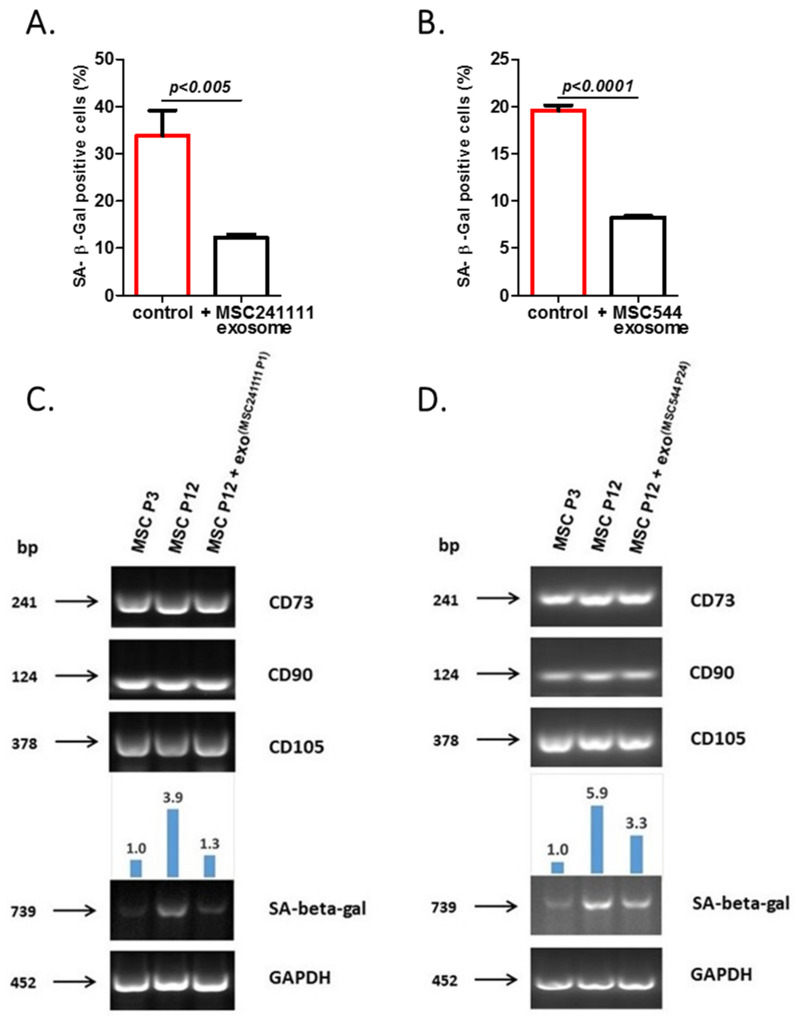
Exosomes isolated from primary young human MSC241111 P1 (**A**) or from continuously proliferating MSC544 (**B**) reduced a senescent phenotype. About 5000 cells of the senescent UC-MSC150819 P12 were plated in wells of a 24-well plate with 1 mL of MSC growth medium. The cells in each well were stimulated either with 50 µL PBS (control) or with 50 µL of PBS-resuspended MSC-derived exosomes with a ratio of 2.6 × 10^5^ exosomes/cell, respectively. After 72 h of incubation, the cells were fixed and stained for SA-β-gal activity. SA-β-gal-positive cells were quantified in representative documentations from 4 independent cultures. Data represent the mean ± s.d. of 4 replicates (*n* = 4), and significance was calculated by unpaired *t*-test. The population bars expressing higher senescence are marked in red. Expression of the MSC markers CD73, CD90, and CD105, and the aging marker SA-β-gal with GAPDH as a control, was compared in young UC-MSC150819 P3 and the same senescent UC-MSC150819 P12 stimulated for 72 h with PBS, and in senescent UC-MSC150819 P12 after a 72 h stimulation with 2.6 × 10^5^ exosomes per cell (+exo). The exosomes were dissolved in PBS and previously isolated from MSC241111 P1 (**C**) and MSC544 P24 (**D**). Densitometry scanning of SA-β-gal expression was normalized to the corresponding GAPDH transcripts, and relative expression levels were inserted as bar graphs.

The data revealed that the growth-arrested and senescent UC-MSC150819 P12 resumed proliferative capacity upon stimulation with MSC-derived exosomes from either young primary MSC241111 P1 by 6% within 72 h (Figure 2, left panel) or from the permanently growing human MSC544 cell line by 46% within 72 h (Figure 2, right panel). The increase in the cell number was statistically significant after 72 h of exosome stimulation, suggesting the capability of MSC-derived exosomes to promote cell growth in an aged population. Consequently, we addressed the question as to whether MSC-derived exosomes also affect a senescent phenotype. The expression of SA-β-gal (synonymously used for GLB1) was examined in aged MSC cultures both at the protein and transcript levels (Figure 3).

Cultures of UC-MSC150819 P12 demonstrated marked expression of the SA-β-gal by 33.8% ± 13.1% (*n* = 4) (Figure 3A, red bar). However, stimulation with exosomes from young primary MSC241111 P1 was associated with a significant reduction in SA-β-gal expression to 12.2% ± 1.7% (*n* = 6) within 72 h (Figure 3A, black bar). These effects were substantiated by a parallel experiment using exosomes from the MSC544 cell line. UC-MSC150819 P12 cultures revealed a SA-β-gal expression of 19.6% ± 1.1% (*n* = 4) (Figure 3B, red bar), while incubation with MSC544 exosomes at a ratio of 2.6 × 10^5^ exosomes/cell demonstrated a significant reduction in SA-β-gal expression to 8.3% ± 0.3% (*n* = 6) within 72 h (Figure 3B, black bar). Together, the data suggested that MSC-derived exosomes either from a young primary MSC culture or from the steady state-growing MSC544 cell line enhance the proliferative capacity of growth-arrested senescent MSC. Simultaneously, these different exosome populations significantly reduced senescence, suggesting the promotion of a rejuvenated phenotype.

The effects of MSC-derived exosomes on the reduction in SA-β-gal activity were further substantiated at the molecular level using differentially aged MSC. The transcript analysis of SA-β-gal was performed in young UC-MSC150819 P3, in the same senescent UC-MSC150819 P12, and in exosome-stimulated senescent UC-MSC150819 P12. The typical MSC markers CD73, CD90, and CD105 were expressed at similar levels in all investigated MSC populations (Figure 3C,D), suggesting little if any functional changes during the senescence of UC-MSC150819 P12. However, SA-β-gal transcripts were significantly increased in senescent UC-MSC150819 P12 by 3.9- (Figure 3C) and 5.9-fold (Figure 3D) compared to young UC-MSC150819 P3 after normalization to the corresponding GAPDH expression levels as a control. Conversely, the stimulation of senescent UC-MSC150819 P12 with exosomes from young MSC241111 P1 markedly reduced the elevated SA-β-gal transcripts back to 1.3-fold within 72 h (Figure 3C). Similarly, the incubation of senescent UC-MSC150819 P12 in the presence of MSC544-derived exosomes was accompanied by a down-modulation of SA-β-gal mRNA expression to 3.3-fold within 72 h (Figure 3D).

**Figure 4:**
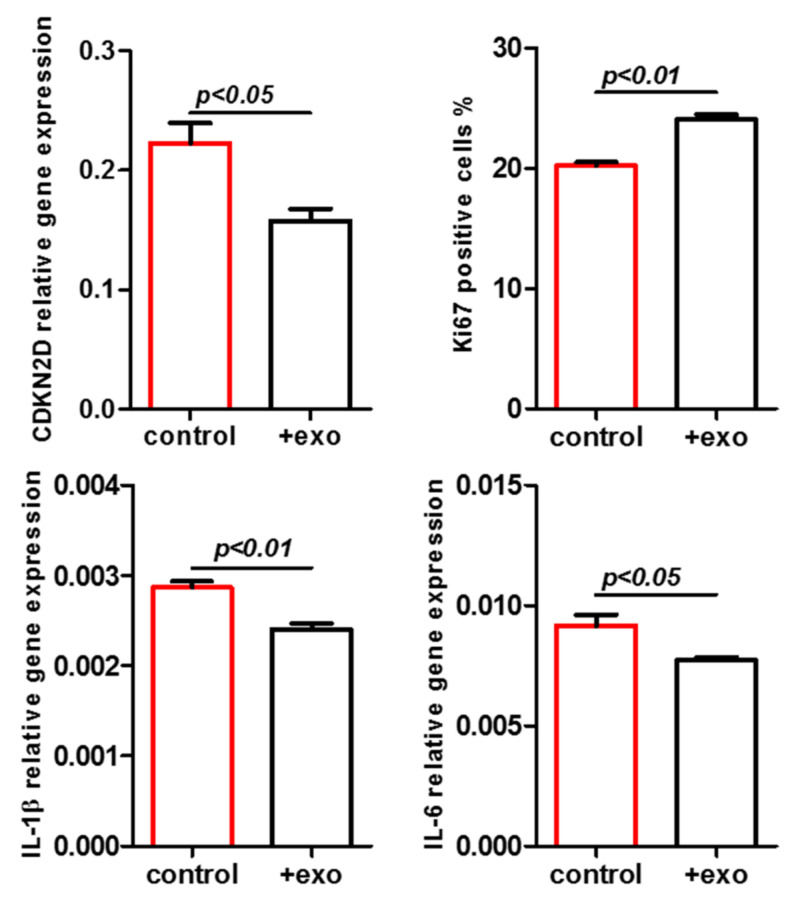
Senescent UC-MSC150819 P12 was treated with PBS (control) or with MSC544-derived exosomes (+exo), expression of proliferation-associated CDKN2D was evaluated (**upper left** panel), and the percentage of Ki67-positive cells was counted (**upper right** panel). Similarly, expression of some SASP-associated markers, including IL-1β (**lower left** panel) and IL-6 (**lower right** panel), was quantified. The population bars expressing higher senescence are marked in red.

To further substantiate this effect of rejuvenation by MSC-derived exosomes, we tested the expression of proliferation-associated and SASP factors in senescent UC-MSC150819 P12 after stimulation with MSC544-derived exosomes in a different approach (Figure 4).

A significantly reduced expression of the cyclin-dependent kinase inhibitor 2d (CDKN2D, p16INK4a) was observed in exosome-stimulated senescent MSC after 72 h (Figure 4, upper left panel). CDKN2D represents a member of the INK4 family of cyclin-dependent kinase inhibitors that contributes to inhibiting CDK4 or CDK6 activation and, consequently, G1 phase cell cycle progression. Moreover, exosome stimulation also markedly elevated the amount of Ki67-positive cells, which further substantiated an increased proliferative capacity of the senescent UC-MSC150819 P12 (Figure 4, upper right panel). Conversely, the expression of some SASP factors, such as IL-1β (lower left panel) and IL-6 (lower right panel), was significantly reduced by the MSC544-derived exosomes.

Together, the effects of MSC-derived exosomes promote proliferative capacity and reduce senescence, representing an important property for tissue repair and regeneration.

**Figure 5:**
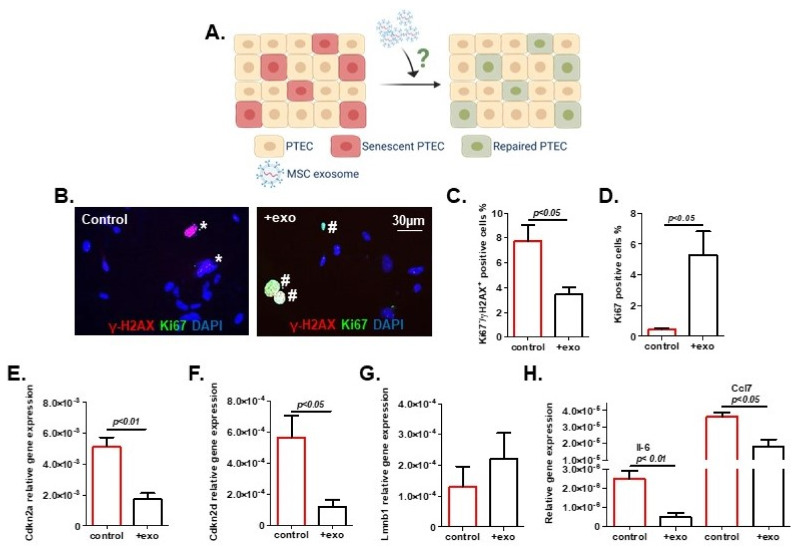
Exosome treatment reduced primary tubular epithelial cell (PTEC) senescence. (**A**) Schematic of experimental procedure (created using BioRender). (**B**) Representative images of PTEC showing immunofluorescent staining for γ-H2AX and Ki67. Cells were counted as senescent (marked with *) when Ki67 negative and simultaneously positive for over five γ-H2AX foci. Ki67-positive cells are marked with #. Percentages of (**C**) senescent cells and (**D**) proliferating cells were quantified. Transcription level of senescent markers: (**E**) Cdkn2a, (**F**) Cdkn2d, (**G**) Lmnb1, and (**H**) senescence-associated secretory phenotype (SASP) markers Il-6 and Ccl7. Exosomes: exo. Results are presented as means ± SEM (*n* = 4). Significances were calculated by *t*-test. The population bars expressing higher senescence are marked in red.

Previous work, particularly with exosomes but not with the larger microvesicles isolated from bone marrow-derived MSC, stimulated proliferative effects in murine tubular epithelial cells [45]. Accordingly, we wanted to test the potential of MSC-derived exosomes to counteract senescence in tubular epithelial cells, the leading kidney cell type. To this end, we used exosomes from young MSC (MSC081113^GFP^ P2) to treat murine primary tubular epithelial cells (PTEC). After the induction of senescence in PTEC by irradiation, exosomes were added using 2.6 × 10^5^ exosomes/cell (Figure 5A), and cultures were evaluated at 72 h. Exosome treatment was associated with a striking rejuvenation of PTEC with a significant reduction in the senescent phenotype. We found less DNA damage, as shown by a significant reduction in γ-H2AX^+^/Ki67^−^ cells after exosome treatment (Figure 5B,C). Concomitantly, we observed a significant increase in proliferating cells as quantified by Ki67 positivity (Figure 5D), and a significantly decreased expression of senescence markers Cdkn2a and Cdkn2d (p19INK4d) (Figure 5E,F). The expression of Lmnb1, on the other hand, showed an upwards trend, which is consistent with a mitigated senescent state (Figure 5G). In agreement with these findings, we observed a reduced expression of Il-6 and Ccl7, suggesting diminished SASP activity (Figure 5H).

The effectiveness of PTEC compared to HuVECs for the uptake of MSC-derived exosomes was tested. Following the addition of 2.9 × 10^3^ GFP-labeled exosomes/cell after isolation from MSC544^GFP^, HuVECs demonstrated about 1% of exosome incorporation after 4 h, whereas PTEC revealed a significant uptake of 8%. This is in line with previous results of MSC-derived exosome uptake by cancer cells within 24 h [40]. Nevertheless, the MSC-mediated activation of endothelial cells plays an important role in neo-vascularization during tissue repair.

## 3. Discussion

Accumulating evidence suggests a crucial contribution of cellular senescence to the age-related deterioration of kidney structure and function [5,6]. Antagonizing the development of senescence and eliminating existing senescent cells have shown promise in ameliorating acute kidney injury (AKI) and chronic kidney disease in different mouse models [3,4,20,46,47]. In particular, MSC and their paracrine release of a heterogenous panel of soluble trophic factors together with EVs contribute to attenuate renal injury, as demonstrated in animal acute kidney injury models, such as renal ischemia-reperfusion injury and drug-induced renal injury [48]. In this report, we found that human MSC-derived exosomes, which are able to exert pro-proliferative and anti-senescent effects on other senescent MSC cultures, are also able to reverse the senescent phenotype of primary tubular cells isolated from murine kidneys. These findings are important as they provide a basis for further research to explore the therapeutic potential of MSC-derived exosomes for the treatment of age-associated kidney and other diseases.

MSC-derived exosomes from young primary MSC could restore proliferative capacity and reduce senescence in growth-arrested and aged MSC of a different donor. This system resembles a type of heterochronic parabiosis, as described in the rejuvenation studies of Conboy et al., with the restoration of notch signaling in aged satellite tissues [49]. In addition to primary cells, exosomes isolated from the proliferating human MSC544 cell line exhibited similar properties to rejuvenate a senescent phenotype. These cells can reversibly switch between a growth-arrested senescent state during prolonged confluency and vice versa, a regained proliferative capacity with reduced aging markers after reculture in a subconfluent environment. Thereby, continuously proliferating MSC544 underf subconfluent conditions releases significantly more exosomes compared to a confluent and senescent MSC544 population [25]. Accordingly, exosomes from proliferating MSC544 provide a long-term reproducible source with constant properties that may represent useful clinical vehicles, e.g., for the successful delivery of chemotherapeutic compounds to cancer cells [43,50].

Previous studies have highlighted the potential of MSC and MSC-derived exosomes in delaying and even reversing kidney disease [51,52] and for improving outcomes after kidney transplantation [53,54]. While these studies have shown immune-modulatory, anti-fibrotic, anti-apoptotic, pro-regenerative and pro-angiogenic properties of MSC or their derivatives, only a few reports are available regarding their potential effects on cellular senescence. Rodrigues et al. demonstrated that human UC-MSC, which were administered intra-peritoneally, protected the kidneys of rats from premature senescence in ischemic acute kidney injury [55]. Kim et al. recently showed that the direct intra-arterial delivery of human adipose tissue-derived MSC can partially alleviate features of senescence after prolonged ischemia in mouse kidneys [56]. We observed a robust reduction in senescence markers in PTEC after treatment with juvenile MSC-derived exosomes. We found reduced levels of γ-H2AX nuclear foci as a sign of diminished DNA damage and reduced expression of cell cycle inhibitors Cdkn2a and Cdkn2d. A restored cell cycle behavior of PTEC was reflected by higher Ki67 positivity and the partial recovery of Lmnb1 expression, a prerequisite for adequate proliferation [57]. Importantly, we also noted a decreased expression of pro-inflammatory cytokines Il-6 and Ccl7, suggesting that MSC-derived exosomes induced an attenuation of SASP-related activity. These findings highlight the potential of achieving similar effects with juvenile MSC-derived exosomes, as previously reported for pharmaceutical senolytics [3,4,5].

As a cell-free system, MSC-derived exosomes offer diminished safety risks, easier dosage and standardization in comparison to the direct use of MSC. Thus, MSC-derived exosomes are considered favorable from a translational perspective. Previous studies have indicated that EVs including exosomes tend to aggregate at the site of injury [58], i.e., labeled exosomes were found to accumulate particularly in the AKI kidney rather than in the healthy control. Moreover, in a recent seminal study, Dorronsoro et al. reported that exosomes released by MSC that were derived from human embryonic stem cells suppressed the senescence of fibroblasts in vitro, leading to the reduced expression of p16INK4a, IL-6 and other markers of senescence [59]. In vivo, they showed that the intraperitoneal injection of exosomes was associated with a reduced senescence load in several organs (including the kidney) and led to healthier aging in progeroid mice. In agreement with these data, our results demonstrated that exosomes derived from neo-natal UC-MSC are potent modulators of senescence in tubular epithelial cells.

Altogether, these data indicated the positive effects of juvenile MSC-derived exosomes in improving aged primary MSC and renal tubular cell senescence.

## 4. Materials and Methods

### 4.1. MSC Culture

Explant cultures for the enrichment of primary human mesenchymal stroma/stem-like cells (MSC) was performed as described for umbilical cord tissue (UC-MSC) [60]. In addition, primary MSC544 was derived from the mammary tissue explants of a patient with a benign phyllodes tumor, as previously described [61]. Briefly, collected tissues were extensively washed with PBS to remove blood cells and cell debris. The tissues were cut into small pieces of approximately 2 mm^3^ and incubated for explant culture. Outgrowing cells with predominant MSC-like morphology were cultivated in MSC growth medium consisting of MSC basal medium (αMEM, 100 U/mL penicillin, 100 µg/mL streptomycin and 2 mM L-glutamine (all from Sigma Chemie GmbH, Taufkirchen, Germany)) supplemented with 10% allogeneic human AB-serum (PAN Biotech GmbH, Aidenbach, Germany), at 37 °C with 5% CO_2_ in a humidified atmosphere. Subculture in passages (P) was performed following treatment of the UC-MSC cultures with accutase (Capricorn Scientific GmbH, Ebsdorfergrund, Germany) and treatment of MSC544 using TrypLE (Life Technologies GmbH, Darmstadt, Germany) at 37 °C for 3 min. The labeling of UC-MSC081113 and MSC544 cultures with GFP was performed by lentiviral transduction using a third generation lentiviral SIN vector containing the GFP gene, as described in [62], to subsequently obtain GFP-labeled exosomes. For the experiments, MSC cultures were used from 4 different donors (young UC-MSC150819 P3 and senescent P12 as cellular models, UC-MSC081113^GFP^ P2, UC-MSC241111 P1 and steady state proliferating MSC544 and MSC544^GFP^ in different passages as the source for exosome preparations).

### 4.2. Preparation and Nanoparticle Tracking Analysis (NTA) of MSC-Derived Exosomes

Exosomes were isolated from three different MSC cultures (MSC081113^GFP^ P2, MSC241111 P1, and from steady state growing MSC544 P17). Subconfluent cultures of each MSC (about 2 × 10^6^ cells) at a density of 1.4 × 10^4^ cells/cm^2^ were washed three times with serum-free MSC basal medium and incubated with serum-free basal medium for a further 24 h. Thereafter, the conditioned medium was removed and sequentially centrifuged in 4 steps (step 1: 360× *g* for 10 min to remove cells; step 2: 2000× *g* for 10 min to remove dead cells; step 3: 10,000× *g* for 30 min to remove debris and large vesicles; step 4: 1,000,000× *g* for 70 min to precipitate exosome-like particles) according to the protocol by Thery et al. [38]. While some characterization for exosomal properties was performed according to the updated MISEV (minimal information for studies of extracellular vesicles) 2018 standards [63], the obtained vesicles were termed exosomes in this manuscript rather than EVs, although no further purification was performed after the 4 centrifugation steps. The precipitated MSC-derived exosomes were resuspended in 50 µL PBS and stored at −80 °C until use for the stimulation of senescent UC-MSC150819 P12 or senescent primary tubular epithelial cells (PTEC) with a ratio of 2.6 × 10^5^ exosomes/cell. NTA of the different exosome preparations in PBS was performed for vesicle concentration, size distribution, and preparation quality in scatter mode using the ZetaView PMX120 NTA (Particle Metrix GmbH, Meerbusch, Germany) with an embedded 40 mW laser at 488 nm and a CMOS camera, as previously described [42].

### 4.3. Immunoblot Analysis of Exosomes

Immunoblot analysis was performed as described previously [64]. Briefly, the protein concentrations of MSC-derived exosome preparations were quantified using the BCA method (Thermo Scientific, Schwerte, Germany), and 20 µg of exosome proteins was separated on a 10% SDS polyacrylamide gel and transferred to a nitrocellulose membrane (GE Healthcare Lifescience, Freiburg, Germany) after semi-dry blotting (Peqlab Biotechnology GmbH, Erlangen, Germany) at 1.5 mA/cm^2^ for 1 h. The blots were incubated with a 1:500 dilution of the mouse monoclonal CD9 antibody (clone Ts9), a 1:250 dilution of the mouse monoclonal CD63 antibody (clone Ts63), a 1:250 dilution of the mouse monoclonal CD81 antibody (clone M38) (all from Invitrogen/Thermo Scientific, Schwerte, Germany) and a 1:200 dilution of the mouse monoclonal GAPDH antibody (clone 6C5, mouse, Santa Cruz Biotechnology Inc., Dallas, TX, USA). The membranes were washed with TBS/Tween-20, and visualization was performed by autoradiography using WesternBright Chemiluminescent Substrate Quantum (Biozym Scientific, Hessisch Oldendorf, Germany).

### 4.4. Senescence Assay

Dual-fluorescence staining for Ki67 and γ-H2AX was used to identify senescent cells (simultaneously Ki67-negative and -positive for more than five γ-H2AX foci). Cells were fixed with paraformaldehyde and stained with anti-Ki67 antibody (Thermo Scientific, Schwerte, Germany) and anti-phospho-histone-H2AX (EMD Millipore, Burlington, MA, USA). Secondary antibodies were Alexa 488 or Alexa 555 (Invitrogen/Thermo Scientific, Schwerte, Germany). The SA-β-gal assay was performed as previously described [65]. Briefly, the different MSC cultures were fixed and stained with the SA-β-gal substrate at pH 5.9 to 6.0 for 24 h/37 °C in the dark according to the manufacturer’s recommendations (Cell Signaling Technology Inc., Danvers, MA, USA). Following two washes with PBS, the documentation of the differentially stained cell cultures was performed by phase contrast microscopy. SA-β-gal-colored cells were randomly chosen and quantified in 4 different cultures.

### 4.5. PTEC Culture, Senescence Induction and Exosome Treatment

Primary tubular epithelial cells (PTEC) were isolated from the kidneys of 3-month-old male C57BL/6J mice as previously described [66]. Kidneys were harvested, minced and digested in bubble-agitated Hanks 199 medium (Gibco/Thermo Scientific, Schwerte, Germany) containing 0.125% Collagenase Type I (Affymetrix Inc., Santa Clara, CA, USA) at 37 °C for 40 min. The solution was then filtered using a 40 µM cell strainer (BD Biosciences Inc., Franklin Lakes, NJ, USA) to separate tubules by size. Tubular fragments were cultured in REGM-II medium (Promocell GmbH, Heidelberg, Germany) for 6 days, and the confluent PTEC culture was exposed to γ-irradiation (10 Gray) to induce senescence. Aliquots of exosomes derived and isolated from human UC-MSC081113^GFP^ P2 were added into PTEC cultures at a ratio of 2.6 × 10^5^ exosomes/cell 8 days after the irradiation. Samples were harvested after 72 h of exosome co-culture, and the senescence load in PTEC was evaluated.

### 4.6. Uptake of MSC-Derived Exosomes by PTEC and Human Umbilical Vein-Derived Endothelial Cells (HuVECs)

Incorporation of GFP-labeled exosomes was performed as previously described [40]. Briefly, GFP-labeled exosomes were isolated from MSC544^GFP^ P26 and quantified by NTA. PTEC and HuVECs as controls were plated in a 96-well plate (Nunc/ThermoFischer Scientific, Roskilde, Denmark) at a density of 4000 cells/well. HuVECs were prepared and cultured as previously described [42]. To allow incorporation, equal aliquots of about 2.9 × 10^3^ GFP-labeled exosomes/cells were added to the PTEC and HuVEC cultures for 4 h. Following three extensive washes with PBS to remove non-incorporated exosomes, quantification of cell-associated exosomes was performed by fluoroscan assay.

### 4.7. Relative Gene Expression by RT-qPCR, RT-PCR

RNA was isolated using NucleoSpin RNA Plus (Machery-Nagel GmbH, Düren, Germany). For cDNA synthesis, 1000 ng of RNA was used for reverse transcription by RNA-dependent DNA Polymerase (Promega Inc., Madison, WI, USA). The levels of mRNA expression were determined by RT-qPCR with specific primers (Cdkn2a: forward- 5′-CGA ACT CTT TCG GTC GTA CCC-3′, reverse- 5′-CGA ATC TGC ACC GTA GTT GAG C-3′, Cdkn2d: forward- 5-TCG TGA ACA TCT TGT TGA GGC TA-3′, reverse- 5-GTT GCC CAT CAT CAT CAT CAC CTG-3′, Lmnb1: forward- 5′-GGG AAG TTT ATT CGC TTG AAG A-3′, reverse- 5-ATC TCC CAG CCT CCC ATT-3′,Il-6: forward- 5-TAG TCC TTC CTA CCC CAA TTT CC-3′, reverse- 5′-TTG GTC CTT AGC CAC TCC TTC-3′ Ccl7: forward- 5-CCT GGG AAG CTG TTA TCT TCA AG-3′, reverse- 5-CCT CCT CGA CCC ACT TCT GA-3′, IL-1β: forward- 5′-ATG ATG GCT TAT TAC AGT GGC AA-3′, reverse- 5′-GTC GGA GAT TCG TAG CTG GA-3′, IL-6: forward- 5′-ACT CAC CTC TTC AGA ACG AAT TG-3′, reverse-5′-CCA TCT TTG GAA GGT TCA GGT TG-3′, CDKN2D: Hs00924091_m1 (Thermo Scientific, Schwerte, Germany)) and RT-PCR with the specific primers for SA-β-gal (synonymously used for GLB1) (forward: 5’-AGG GAG TCC TTG AGC GAA AC-3’, reverse: 5’-AGG GAG GAT CTG TGA GGT TAG T-3’, transcript 739bp). For RT-qPCR, relative mRNA levels were calculated according to the 2^−ΔCt^ methods for all genes tested. The β-actin housekeeping gene (forward: 5′-CCT CTA TGC CAA CAC AGT-3′, reverse- 5′-CAT CGT ACT CCT G CT TGC TG -3′) and the GAPDH (forward: 5′-ACC ACA GTC CAT GCC ATC AC-3′, reverse: 5′-TCC ACC ACC CTG TTG CTG TA-3′, transcript 452 bp) expression levels were used as controls, respectively, to normalize the data.

### 4.8. Statistical Analysis

Results are expressed as the means ± SEM. Statistical significance between means was calculated by an unpaired *t*-test (GraphPad Software version 7). *p* < 0.05 was considered statistically significant.

## 5. Conclusions

The encouraging results of this study emphasize the therapeutic property of MSC exosomes in kidney senescence and unveil the potential of a cell-free approach in senotherapy. Given that human MSC can be expanded in bioreactors to generate high yields of MSC-derived exosomes, there is promise for the treatment of age-related kidney pathologies. Moreover, the various trophic compounds delivered by juvenile MSC-derived exosomes provide an orchestrating therapeutic platform to potentially ameliorate further age-related diseases. This may also contribute to reduced senescence and/or promote rejuvenation in various other tissues and organs.

## Figures and Tables

**Table 1:** NTA of MSC-derived exosomes.

MSC Cell Type	Exosome Size (nm)	Amount of Exosome Release per Cell (MSC) in 24 h	Calculated Protein Amount per Exosome (pg/Exosome)
MSC081113^GFP^ P2	177.1 ± 82.3	3.3 × 10^3^	5.03 × 10^−4^
MSC241111 P1	156.1 ± 120.8	1.9 × 10^4^	4.61 × 10^−3^
MSC544 P17	134.7 ± 58.4	1.3 × 10^4^	2.01 × 10^−3^

## Data Availability

The data that support the findings of this study are available from the corresponding author upon reasonable request.

## Abbreviations

EVs	extracellular vesicles
HuVECs	human umbilical vein-derived endothelial cells
(UC)-MSC	(umbilical cord-derived) mesenchymal stroma-/stem-like cells
NTA	nanotracking analysis
PTEC	primary tubular epithelial cells
ROS	reactive oxygen species
SA-β-gal	senescence-associated beta-galactosidase (GLB1)
SASP	senescence-associated secretory phenotype
STASIS	stress or aberrant signaling-inducing senescence
TEM	transmission electron microscopy
